# The Q705K and F359L Single-Nucleotide Polymorphisms of NOD-Like Receptor Signaling Pathway: Association with Chronic Pancreatitis, Pancreatic Cancer, and Periodontitis

**DOI:** 10.1007/s00005-015-0355-9

**Published:** 2015-08-08

**Authors:** Andrzej Miskiewicz, Grzegorz Szparecki, Marek Durlik, Grażyna Rydzewska, Ireneusz Ziobrowski, Renata Górska

**Affiliations:** Department of Periodontology, Medical University of Warsaw, Miodowa 18, 00-246 Warsaw, Poland; Department of Pathology, Medical Univeristy of Warsaw, Warsaw, Poland; Department of Gastroenterological and Transplant Surgery, Central Clinical Hospital, Ministry of Internal Affairs, Warsaw, Poland; Department of Gastroenterology, Central Clinical Hospital, Ministry of Internal Affairs, Warsaw, Poland

**Keywords:** Inflammasomes, Innate immunity, Inflammation mediators, Pancreatic cancer, Chronic pancreatitis, Periodontitis

## Abstract

The aim of this study was to establish the correlation between the occurrence of Q705K and F359L polymorphisms in patients diagnosed with pancreatic diseases and periodontal conditions of various degrees of severity. The above-mentioned genetic markers were assessed in patients with pancreatic cancer (*n* = 18) and chronic pancreatitis (*n* = 39) as well as in a healthy control group (*n* = 115). The established inclusion criteria were the following: Caucasian descent, non-smoking, and age range 20–80, with different levels of periodontitis activity according to S. Offenbacher’s scale. The genotyping reactions were performed by means of an RT-PCR with the use of TaqMan^®^ genotyping assay. Results of the study revealed that the state of periodontium was significantly worse in patients with chronic pancreatitis. The Q705K and F359L polymorphisms were associated with more advanced cases of periodontitis measured by clinical attachment level, whereas the Q705K was associated with intensified bleeding index. Furthermore, the F359L single-nucleotide polymorphism was significantly higher in the group with chronic pancreatitis (*p* < 0.0001; OR = 6.8571). Whereas, the prevalence of Q705K polymorphism was higher in the group of pancreatic cancer (*p* = 0.107; OR = 3.3939). This study suggests that the exaggerated inflammatory response provoked by Q705K and F359L might be the common denominator for periodontitis, pancreatic cancer, and chronic pancreatitis. These findings might constitute the basis for a new diagnostic and therapeutic approach.

## Introduction

**Fig. 1:**
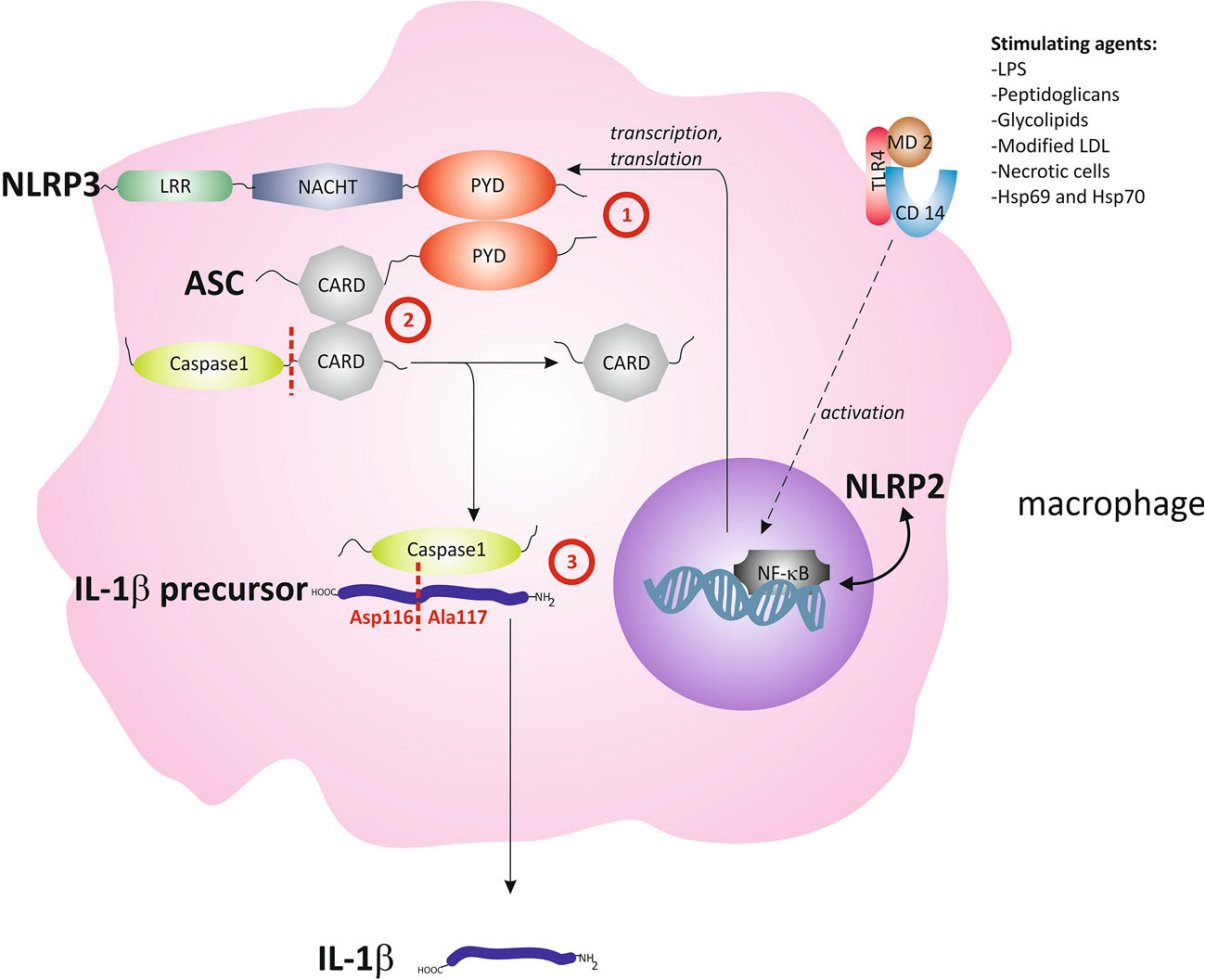
The inflammasome axis. The NLRP3 inflammasome is composed of NLRP3, ASC, and procaspase-1. Production of these proteins is regulated by NF-κB transcriptional factor, which in turn is stimulated by the activation of the TLR4 receptor. Please note that various factors associated with bacterial colonization act as ligands of this receptor. The interaction between the PYD and CARD domains (1 and 2) within the inflammasome leads to caspase-1 activation and cleavage of IL-1β precursor to produce an active form of IL-1β (3)

NOD-like receptor (NLRP) signaling pathway is the phylogenetically oldest sequence of pro-inflammatory gene expression and activation of the encoding proteins. The human *NLRP2* and *3* genes encode a crucial regulatory factor responsible for an increased transcription of chemokine 8 (CXCL-8), interleukin (IL)-18, and IL-1β (Hajishengallis 2014; Hu et al. 2014). Moreover, the cytoplasmic NLRP3 acting on their precursor is responsible for an enzymatic activation of cytokines (see Fig. 1). Recent in vitro reports state that the change of one amino acid in NLRP3: Q705K (glutamine replaced with lysine) is responsible for an increased enzymatic cleavage of pro-IL-1β into a fully active form (Verma et al. 2012). On the other hand, the F359L mutation in NLRP2 (threonine is replaced by methionine in this case) is associated with a higher rate of pro-inflammatory gene expression (*CXCL*-*8, Il*-*18, and Il*-*1β*) (Belibasakis and Johansson 2012; Fontalba et al. 2007; Verma et al. 2012). The most important triggering factor of an increased expression and activation of cytokines mentioned above is the stimulation of CD14 receptor mainly by bacterial antigens. Therefore, the aim of this study was to evaluate periodontal status in reference to a single-nucleotide polymorphism (SNP) of NLRP2: F359L (*rs17699678* C>T) and NLRP3: Q705K (*rs35829419* C>A) in patients with pancreatic cancer and chronic pancreatitis as well as in the control group.

At the ASCO meeting, Wong et al. (2009) suggested that the presence of specific transcriptomic and microbiological markers might be useful in detecting pancreatic cancer. Moreover, Zhang et al. (2010) confirmed the hypothesis that an expression of *KRAS* and other genes (*MDBL*, *ACRV1*, and *DPM1*) is elevated in unstimulated saliva in patients with pancreatic cancer. The above findings support growing evidence that oral status, bacterial colonization, and the presence of specific mRNA in saliva may be useful in diagnosing pancreatic conditions. However, no evaluation of the periodontal status in patients with pancreatic cancer and chronic pancreatitis has been made. Despite the noted progress and much research carried out, the pathogenesis of periodontitis, pancreatic cancer, and chronic pancreatitis has not yet been fully explained. However, it was discovered that conditions debilitating excretory functions of the pancreas modulate the course of periodontitis (Camen et al. 2012). Furthermore, periodontitis and gingivitis may constitute the first visible sign of ongoing diabetes caused by chronic pancreatitis, pancreatic cancer, or in case of comorbidity of the two last conditions. On the molecular and biochemical levels, these changes may manifest in saliva composition and crevicular fluid changes (Yoon et al. 2012). Conditions undermining immunity and promoting an inflammatory process disrupt defense mechanisms of the host, and monitoring the components of unstimulated saliva might provide a valuable diagnostic tool which in turn represents an ongoing systemic condition.

The scientific novelty of our study lies in transposing the results of the available in vitro studies (Fontalba et al. 2007; Verma et al. 2012) into clinically significant conditions of patient populations remaining under the care of health professionals. Moreover, the current study introduces research on a group of patients with conditions that have never been analyzed before in the context of *NLRP2* and *3* polymorphisms: periodontitis, chronic pancreatitis, and pancreatic cancer. Our study paves the way for facilitating an early detection of latent pancreatic cancer, especially in the course of chronic pancreatitis. Nowadays, the medical professionals search for methods helping differentiate between chronic pancreatitis alone and comorbid pancreatic cancer in chronic pancreatitis. This is one of the most crucial problems in modern gastroenterology and the reason for that being the fact that morphological symptoms and clinical signs such as ultrasonography imaging and chronic abdominal pains may successfully hide an adenocarcinomatous process developing secondary to chronic pancreatitis.

Furthermore, the common inflammatory basis, recurring throughout the whole organism, has already given a target point for various therapeutic interventions. The current study, emphasizing immunological findings in pancreatic cancer, might open new areas for intervention in terms of inflammasome activity with their possible clinical implications.

## Materials and Methods

### Study Population

Our study was conducted based on three groups of individuals. The first one (*n* = 18) were patients diagnosed with pancreatic cancer in stages I and II (resectable stages of pancreatic cancer) using routine imaging techniques. They were under the care of Gastroenterological and Transplant Surgery ward of the Central Clinical Hospital of the Ministry of Internal Affairs in Warsaw, Poland. The age of the patients ranged 41–76 years (median 57.5). All of them were examined for our study prior to the planned surgery. The patients came from all parts of Poland and were referred to our Clinical Hospital as a result of a clinical diagnosis of pancreatic cancer which was made following imaging examinations confirmed subsequently by a histopathological examination performed after the surgery. All patients were admitted to our ward within 1 week from the date of referring them to our Clinical Hospital and underwent a periodontal examination on the first day of their stay in our ward. The elicited anamnesis has shown that all the patients of this investigated group demonstrated signs and symptoms (such as chronic pains observed in the abdomen after a meal) that could indicate chronic pancreatitis in progress for at least the past 3 months. However, the group in question was heterogeneous with regard to an earlier diagnosis of chronic pancreatitis as well as alcohol abuse. We excluded patients who took medications which might influence bacterial status of the oral cavity (antibiotics and cytostatics) and/or were edentulous. The use of analgesics and supplementation of pancreatic enzymes were not a criterion for exclusion.

The second group (*n* = 39) were patients of the Gastroenterology ward and the Internal Medicine and Endocrinology ward of the hospital mentioned above diagnosed with chronic pancreatitis. The diagnosis was made using laboratory tests and imaging techniques. Twenty-five patients included in the study were under permanent observation by the Gastroenterology ward and presented to the ward to monitor their disease. Moreover, as they reported to the hospital they did not mention any exacerbation of signs and symptoms related to chronic pancreatitis. The above patients had controlled diabetes (including other types of diabetes) and had previously been recommended to refrain from alcohol which could have indicated an existing alcohol problem. Fourteen patients of the investigated group suffered from cases of biliary stone disease (*cholelithiasis*). They were admitted to hospital due to cases of exacerbated chronic pancreatitis. The age of the patients ranged 21–57 years (median 49.8). From this group, we excluded patients with full prosthetic devices and/or pancreatitis secondary to cystic fibrosis.

The third group consisted of volunteers (*n* = 115). Criteria for inclusion were as follows: no pathological changes in pancreas including chronic pancreatitis, nor in biliary ducts as shown in the ultrasonography examination of the abdomen performed prior to the study. Patients included in this investigated group originated from the ward of Internal Medicine and Endocrinology who at the moment of conducting the study were hospitalized at the ward and/or reported to the out-patient clinic. The most common reasons for reporting to the ward were: *incidentaloma*—hormonally inactive adrenal gland diseases, Hashimoto’s auto-immune thyroid disease, primary and secondary hypertension, anemias, and heart arrhythmias. Criteria for exclusion were as follows: alcohol abuse, tobacco smoking, oral hypoglycemic agents’ use, diabetes, aggressive periodontitis, edentulous subject, and age below 20 or over 80 years. The age of the volunteers ranged 20–80 years (median: 45.8).

The authors have based their criteria for recruiting patients to the investigated groups on the experiences described in previously published studies (Kuruma et al. 2014; Lin et al. 2013; Nakao et al. 2011). The experiments were undertaken with the understanding and written consent of each subject. Furthermore, the study was independently reviewed and approved by the Board of Ethics appointed by the Dean of the Warsaw Medical University. All individuals enrolled in the study agreed to participate in it by signing an informed consent form. The study was carried out from October 2011 until January 2015.

### Periodontal Examination

First, a medical anamnesis was elicited from the study patients concerning their previous dental history. The emphasis was put on the periodontal treatment including scaling, root planning procedures, and surgical removal of teeth which were qualified as a potential source of a systemic infection. Afterwards, all individuals were examined using the WHO 621 standardized periodontal probe. Measurements were taken at the four sides of the tooth: mesiobuccal, midbuccal, distobuccal, and midlingual. The following indices were assessed:

1. Bleeding index, measured as bleeding on probing (BOP) at four sites of each tooth (%), according to Ainamo’s scale;
2. Probing depth (PD), distance between gingival margin and the pocket’s bottom, measured at four sites of each tooth;
3. The loss of clinical attachment level (CAL) being the distance measured from the base of periodontal pocket and the cemento-enamel junction.

The selected periodontal indices reflect the periodontal status of the probands included in the study. The periodontal index-CAL defines the degree of the advancement of the morphological lesions in the periodontium caused by the loss of the alveolar bone in the maxilla and mandible (Vandana and Gupta 2009). However, the BOP and the PD indices define the inflammation in progress in the area of the periodontal tissues. Furthermore, the activity of periodontitis was assessed using a new and comprehensive five-grade scale of periodontitis according to Offenbacher et al. (2008). The measurements were rounded off to the nearest millimeter. All the assessments made were noted in the documentation and analyzed, and on that basis the final diagnosis was made. Periodontal examination was conducted by a calibrated dentist.

### SNPs’ Selection in Analyzed Genes and Bioinformatics Analysis

The polymorphisms used for the study were selected based on up-to-date publications analyzing the influence of nucleotide changes introduced by them with regard to their function of NLRP2 and 3 (Cummings et al. 2010; Fontalba et al. 2007; Verma et al. 2012; Yang et al. 2014; Zhang et al. 2011). In order to check the feasibility of analysis of rs17699678 *NLRP2* SNP, we used the database from the HapMap project for the Utah residents with ancestry from northern and western Europe (CEU) as this population appears to be the closest to our research group (Polish patients). For the SNPs proposed in this study, the minor allele frequency was 12.9 %. The same database was used to confirm that the SNP has a probable effect on the NACHT domain of the NLRP2 protein which is a part of the NOD-like receptor. Furthermore, we checked the Short Genetic Variations database in order to confirm that the polymorphism in question causes missense mutation (threonine is replaced by methionine in this case).

Another SNP selected for the study, rs35829419 in *NLRP3*, was evaluated using the same tools and criteria. Minor allele frequency in CEU population in the HapMap project was 5.8 %. This polymorphism has also been associated with the missense mutation (glutamine replaced with lysine).

### Genotyping

The authors of this study collected the material for genomic DNA isolation with the use of a buccal swab taken from every patient enrolled in the study. Samples of the collected material were used to perform genotyping in order to detect SNP of *NLRP2* (F359L) rs17699678 C>T and *NLRP3* (Q705K) rs35829419 C>A. The genotyping reactions were performed by means of real-time polymerase chain reaction (PCR) with the use of TaqMan^®^ genotyping assays. The data obtained from the genotyping in the studied groups were correlated with those in the control group. The molecular part of the proposed project was carried out at the Department of Histology and Embryology in the Center for Biostructure Research of the Medical University of Warsaw which avails of the proper equipment indispensable for conducting the above-mentioned molecular research.

### Statistical Analysis

The comparisons between the groups were performed using the Kruskal–Wallis ANOVA test (in case of comparison of all three groups—this applied to clinical markers of periodontitis) and the Mann–Whitney *U* test (in case of comparison of patients with cancer vs. the rest of the individuals). The association between the categorical variables and the quantitative clinical variables was calculated using the two-way factorial ANOVA. The correlation between the results and the periodontium quantitative indices was calculated using the multilinear regression. The software used was Statistica 10 (StatSoft, USA). *P* values <0.05 were deemed statistically significant.

## Results

The results obtained showed the distribution of two polymorphisms of *NLRP2* (rs17699678 C>T) and *NLRP3* (rs35829419 C>A) genes in Polish subjects with pancreatic diseases and periodontitis. We studied the associations of different alleles with pancreatic cancer, chronic pancreatitis, and periodontitis.

The frequency of *NLRP2* alleles and genotypes was not significantly different between controls and patients with periodontitis; however, the frequency of polymorphic allele T was substantially higher in patients with chronic pancreatitis than in controls with no pancreatic pathology [30.77 vs. 6.09 %, *p* < 0.0001, odds ratio (OR) = 6.8571, 95 % CI = 3.3262–14.1364] see Tables 1, 2, and 3. Similar associations were detected as far as the polymorphic allele positivity is concerned (48.71 vs. 11.40 %, *p* = 0.0001 OR = 7.4538, 95 % CI = 3.1767–17.49). On the other hand, no significant differences in the genotype and in the allele frequency and polymorphic positivity were detected for pancreatic cancer.

**Table 1:** Periodontal disease advancement measured by CAL, BOP, and PD mean values in association with investigated SNPs’ allele frequencies

Pancreatic condition	Periodontal disease parameters			Allele distribution of investigated SNPs		Control vs. investigated group					
						NLRP2			NLRP3		
	CAL (mm)	PD (mm)	BOP (%)	F359L (%)	Q705K (%)	*p* value	OR	OR 95 % CI	*p* value	OR	OR 95 % CI
Cancer	2.4	2.68	16.6	5.56	8.33	N/S	N/S	N/S	0.107	3.3939	0.8095–14.2292
Chronic pancreatitis	3.6	4.08	60.76	30.77	0	<0.0001	6.8571	3.3262–14.1364	N/S	N/S	N/S
Control group	1.62	3.69	26.69	6.09	2.61	N/A	N/A	N/A	N/A	N/A	N/A

Please note the close statistical significance of NLRP3 SNP occurrence in cancer patients

*N/S* statistically insignificant result, *N/A* not applicable

**Table 2:** Association analysis of rs17699678 C>T polymorphism in NLRP2 with periodontitis in terms of allele and genotype distribution

NLRP2—periodontitis	Controls		Chronic periodontitis		Control vs. chronic periodontitis	
	%	*n* = 26	%	*n* = 145	*p* value	OR (95 % CI)
Genotype						
CC	92.30	24	78.62	114	NS	NA
CT	3.85	1	17.24	25	NS	NA
TT	3.85	1	4.14	6	NS	NA
Allele frequency						
C	94.23	49	87.24	253	NS	NS
T	5.77	3	12.76	37	NS	NS
Polymorphic allele positivity						
T+	7.70	2	21.38	31	NS	NS
T−	92.30	24	78.62	114	NS	NS

*NS* statistically insignificant result, *NA* not applicable

**Table 3:** Association analysis of rs1769967 C>T polymorphism in NLRP2 with pancreatic diseases in terms of allele and genotype distribution

NLRP2—pancreas condition	Controls		Cancer		Chronic pancreatitis		Control vs. cancer		Control vs. chronic pancreatitis	
	%	*n* = 115	%	*n* = 18	%	*n* = 39	*p* value	OR (95 % CI)	*p* value	OR (95 % CI)
Genotype										
CC	88.70	102	94.44	17	51.28	20	NS	NA	*p* = 0.00021	NA
CT	10.43	12	0.00	0	35.90	14	NS	NA	*p* = 0.00021	NA
TT	0.87	1	5.56	1	12.82	5	NS	NA	*p* = 0.00021	NA
Allele frequency										
C	93.91	216	94.44	34	69.23	54	NS	NS	*p* < 0.0001	6.8571 (3.3262–14.1364)
T	6.09	14	5.56	2	30.77	24	NS	NS	*p* < 0.0001	6.8571 (3.3262–14.1364)
Polymorphic allele positivity										
T+	11.30	13	5.56	1	48.71	19	NS	NS	*p* = 0.0001	7.4538 (3.1767–17.49)
T−	88.70	102	94.44	17	51.28	20	NS	NS	*p* = 0.0001	7.4538 (3.1767–17.49)

Please note the association of allele T with chronic pancreatitis

*NS* statistically insignificant result, *NA* not applicable

The *NLRP3* polymorphism analyzed in the study was not associated with chronic periodontitis. However, allele A occurred more frequently with patients diagnosed with pancreatic cancer (heterozygotes) as compared to the control group’s probands and patients diagnosed with chronic pancreatitis (Table 1). Despite that fact, the occurrence of mutation Q705K with cancer patients [*p* = 0.107, OR = 3.3939 (0.8095–14.2292)] was not proven to be of statistical relevance (see Tables 4, 5).

**Table 4:** Association analysis of rs35829419 C>A Q705K polymorphism in NLRP3 with periodontitis in terms of allele and genotype distribution

NLRP3—periodontitis	Controls		Chronic periodontitis		Control vs. chronic periodontitis	
	%	*n* = 26	%	*n* = 145	*p* value	OR (95 % CI)
Genotype						
CC	96.15	25	94.48	137	NS	NA
CA	3.85	1	5.52	8	NS	NA
AA	0.00	0	0.00	0	NS	NA
Allele frequency						
C	98.07	51	97.24	282	NS	NS
A	1.92	1	2.76	8	NS	NS
Polymorphic allele positivity						
A+	3.85	1	5.52	8	NS	NS
A−	9615	25	9448	137	NS	NS

*NS* statistically insignificant result, *NA* not applicable

**Table 5:** Association analysis of rs35829419 C>A polymorphism in NLRP3 with pancreatic conditions in terms of allele and genotype distribution

NLRP3—pancreas condition	Controls		Cancer		Chronic pancreatitis		Control vs. cancer		Control vs. pancreatitis	
	%	*n* = 115	%	*n* = 18	%	*n* = 39	*p* value	OR (95 % CI)	*p* value	OR (95 % CI)
Genotype										
CC	94.78	109	83.33	15	100.00	39	NS	NA	NS	NA
CA	5.22	6	16.67	3	0.00	0	NS	NA	NS	NA
AA	0.00	0	0.00	0	0.00	0	NS	NA	NS	NA
Allele frequency										
C	97.39	224	91.67	33	100.00	78	NS*	NS*	NS	NS
A	2.61	6	8.33	3	0.00	0	NS	NS	NS	NS
Polymorphic allele positivity										
A+	5.22	6	16.67	3	0.00	0	NS	NS	NS	NS
A−	94.78	109	83.33	15	100.00	39	NS	NS	NS	NS

Allele A occurs more often in patients with pancreatic cancer

*NS* statistically insignificant result, *NA* not applicable

* *p* = 0.107, OR = 3.3939 (0.8095–4.2292)

All periodontal parameters (BOP and CAL) were significantly worse (*p* = 0.001 and *p* = 0.001, respectively) in patients with chronic pancreatitis than in two other groups (Table 1). In patients with pancreatic cancer, a moderate and early stage of periodontitis was most common—BGI-P1 and P2 were noted in 33 and 11 % of cases, respectively, whereas gingivitis was observed in 27 % of individuals. The mean pocket depth was 2.68 ± 0.74 mm, BOP 16.6 %, and CAL 2.4 mm. In the control group the BGI-G—gingivitis was observed in 6 % of cases, whereas BGI-P1 and P2 were found in 31 and 43 % of cases, respectively, wherein the mean pocket depth was 3.69 ± 1.09 mm and BOP 26.69 %. Mean CAL amounted to 1.62 ± 1.02 mm (Table 1). On the other hand, periodontitis was observed in all patients with chronic pancreatitis. In 77 % of cases, the BOP index was more than 50 % and the mean depth of the periodontal pocket was 4.08 ± 0.83 mm indicating severe periodontitis BGI-P3 and CAL 3.6 mm.

**Fig. 2:**
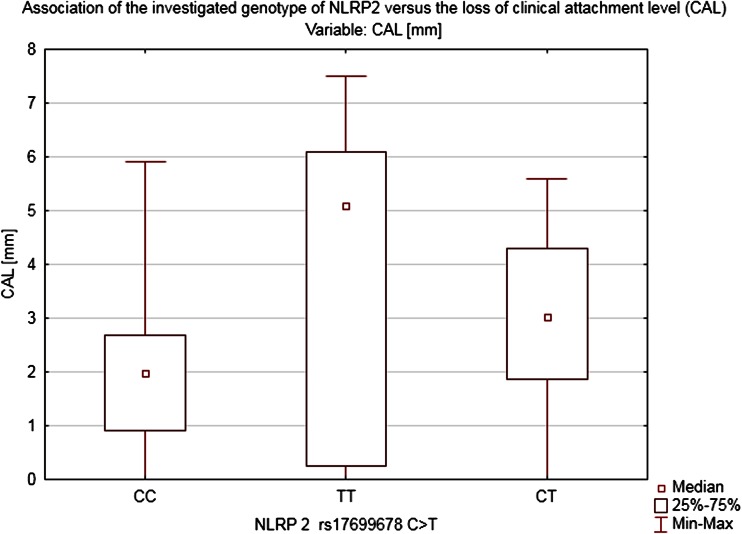
Association of the investigated genotype of NLRP2 vs. the loss of CAL

**Fig. 3:**
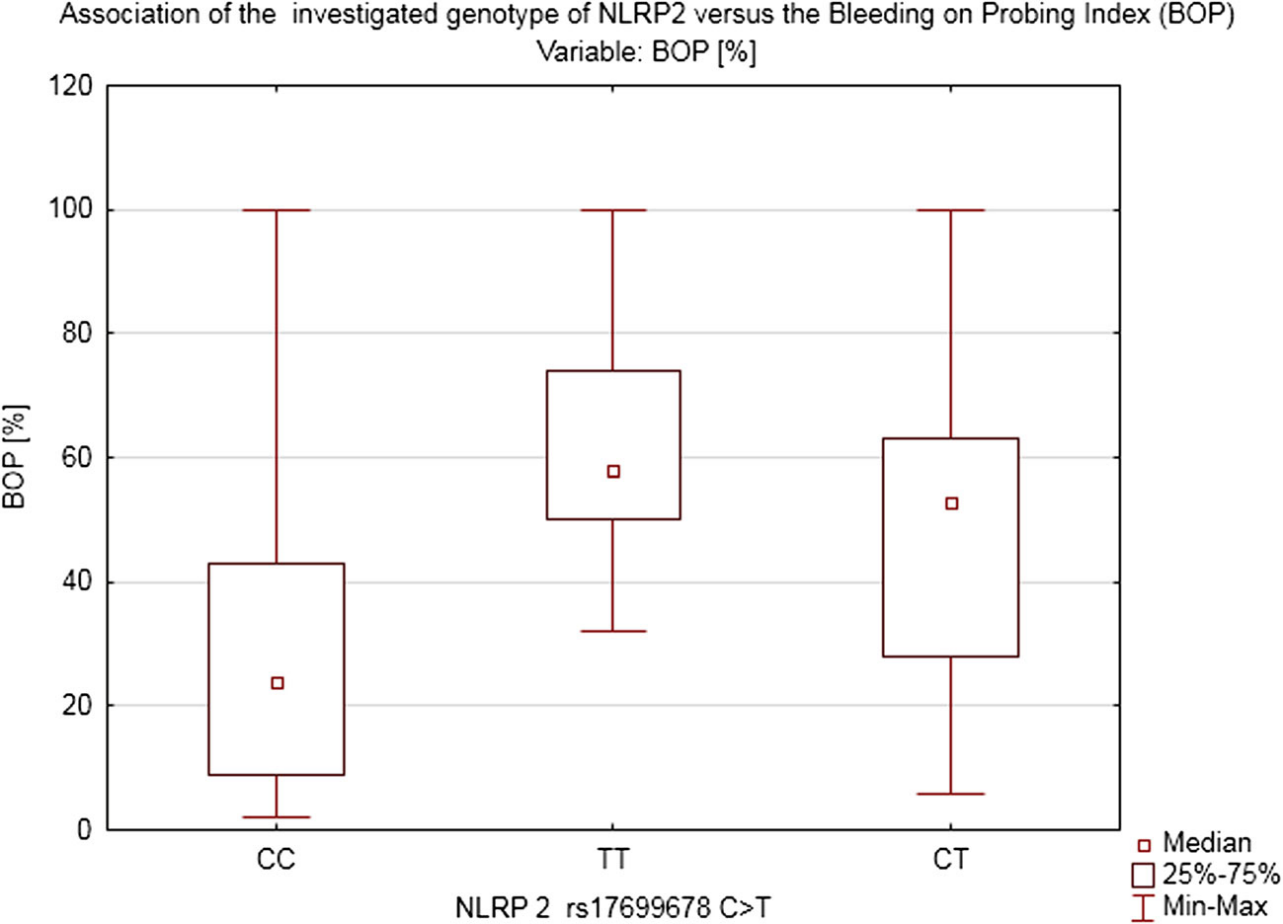
Association of the investigated genotype of NLRP2 vs. the BOP index

Both polymorphisms analyzed in this study had a significant impact on the state of periodontium. As far as NLRP2 is concerned, the genotype seems to have been correlated with all three parameters taken into account in our study. Homozygotes *TT* and heterozygotes *CT* evinced definitely worse condition of periodontium as measured in terms of BOP = 52.75 ± 27.07 % (*H* = 22.55056, *p* < 0.0001), CAL = 3.32 ± 1.94 mm (*H* = 15.89681, *p* = 0.0004), and mean pocket’s depth PD = 4.11 ± 0.93 mm (H = 7.480116, *p* = 0.0238) than homozygotes *CC*: BOP = 28.05 ± 0.93 %, CAL = 1.87 ± 1.31 mm, and PD = 3.58 ± 1.09 mm (Figs. 2, 3). Despite that fact, no statistically relevant link between polymorphism NLRP2 and a specific type of the periodontal inflammation has been shown.

**Fig. 4:**
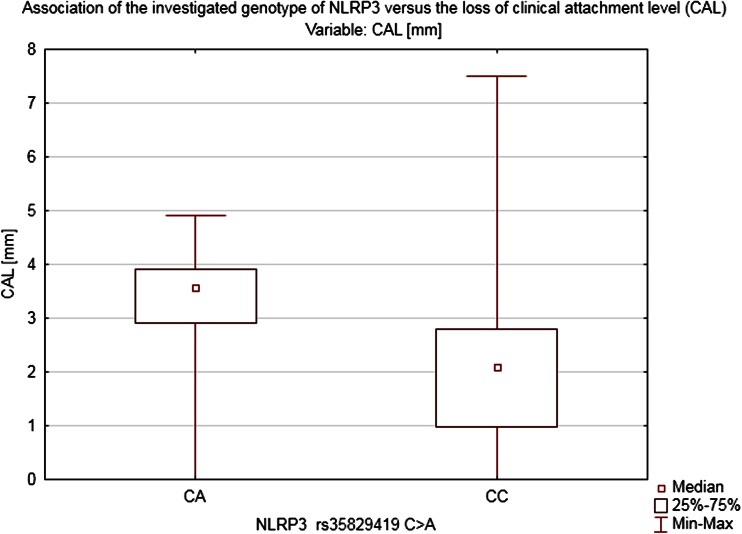
Association of the investigated genotype of NLRP3 vs. the loss of CAL

**Fig. 5:**
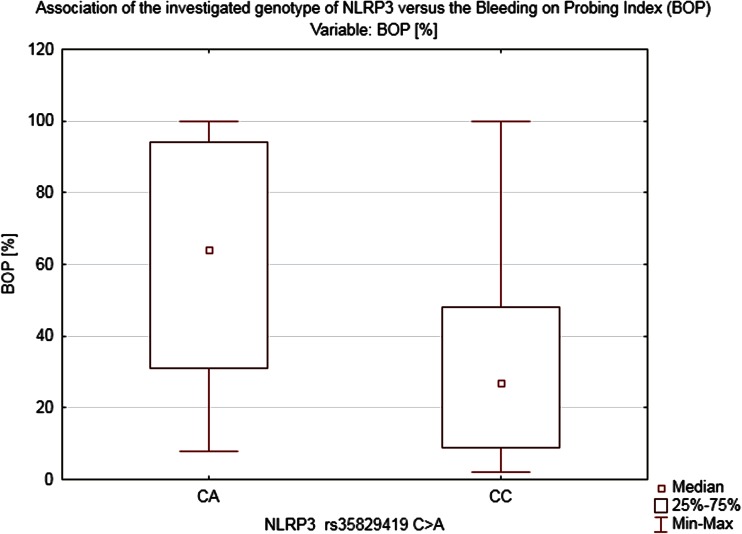
Association of the investigated genotype of NLRP3 vs. the BOP index

Heterozygosity in NLRP3 (*CA*) turned out to be a strong factor that could be linked to more pronounced bleeding on probing BOP = 61.22 ± 18.69 % (*Z* = 2.5339, *p* = 0.009) and higher CAL = 3.71 ± 0.81 mm (*Z* = 2.6749, *p* = 0.006) (Figs. 4, 5). On the other hand, the pocket’s depth was not found to have been influenced by the genotype in the locus mentioned above in our study (PD = 3.3 ± 1.25 mm).

## Discussion

Our study demonstrated that the prevalence of rs35829419 (Q705K, NLRP3) polymorphism is higher in subjects with pancreatic cancer and that of rs17699678 (F359L, NLRP2) polymorphism is higher in the group of chronic pancreatitis. We have found no correlation between the genotype of *NLRP2* and *NLRP3* and the final periodontal diagnosis. We partially confirmed our hypothesis put forth in the introduction that the mentioned polymorphisms might be susceptibility markers for pancreatic cancer, chronic pancreatitis, and periodontitis. However, the etiopathology of these conditions has not yet been fully explained. Their pathophysiology includes activation of specific pathways of inflammatory genes’ expression such as the described axis: CD14—NLRP2 and NLRP3—fully active cytokines. The most important among them is the IL-1β which plays a key role in the studied conditions. We presume that the lack of any correlation between genotype and periodontitis in the studied groups results from a morbidity rate of the studied conditions. Periodontitis is recognized as a population disease, whereas pancreatic cancer and chronic pancreatitis occur in the population at the levels of 4.35–5.5 and 14.9 cases, respectively, per 100,000 (De-Las-Heras-Castano 2014; Maitra and Hruban 2008; Yadav and Lowenfels 2013; Yadav et al. 2011). Although pancreatic cancer has a lower incidence in Poland, it remains the fourth most common cause of death in the USA. Moreover, recurrent episodes of acute pancreatitis predispose patients to chronic pancreatitis. Additionally, cancer may develop in patients with chronic pancreatitis. Pancreatic adenocarcinoma in the course of chronic pancreatitis gives the same clinical signs as chronic pancreatitis alone, such as chronic abdominal pain, frequent exacerbations of chronic pancreatitis, and endocrine dysfunction. Therefore, new markers that could facilitate making a final diagnosis of a given medical condition should be searched for and further investigated into. Hopefully, the present study lays a good foundation for further research to be conducted based on the associations between polymorphisms of NLRP and chronic pancreatitis and pancreatic cancer.

Although the study showed no clear association between the two analyzed SNPs and the diagnosis of periodontitis in the studied population, there was indeed a distinct correlation between the investigated polymorphisms and the selected periodontal parameters: BOP and CAL. Since we used the probing depth (PD) as the basic parameter of our study representing the current periodontal inflammatory status (also used as the basic parameter in the S. Offenbacher’s scale) and CAL which indicates a long-term advancement of the disease and shows advanced morphological lesions in the periodontium (AAP classification), our study revealed no correlation whatsoever between the studied SNPs and the PD. The above, therefore, cannot be used as a basis for making the right diagnosis regarding the detected polymorphisms. It is worth noting that in patients with *NLRP2* polymorphism the bleeding on probing index was high (BOP = 52 %) and in patients with *NLRP3* polymorphism as well (BOP = 61.2 %). As already demonstrated by in vitro studies, both of the investigated SNPs might contribute to exacerbating an inflammation process. The above was indeed confirmed by our clinical study; we demonstrated a clear link between Q705K polymorphisms and intensified bleeding which alone points to a case of active periodontitis.

The degree of the periodontal inflammation advancement measured by the CAL parameter is clinically relevant. The authors of the study demonstrated that in the group of patients diagnosed with the mutation NLRP2 and NLRP3 as well as in chronic pancreatitis group the mean CAL index was more than 3 mm regardless of the PD, whereas in healthy individuals who are not carriers of the muted alleles CAL amounted to 1.62 mm. The above correlation may indicate an overactive phenotype of the investigated groups’ patients in whom the presence of the modifiable risk factors occurs. The study carried out has shown that in the group of heterozygotes *CA* (NLRP3) there was an incommensurably high bleeding index >60 % with a moderate probing depth of 3.3 mm. Moreover, in both investigated groups *CA* (NLRP3), *TT*, and *CT* (NLRP2), an accelerated loss of the CAL >3 mm was detected which is a long-term marker of the periodontium’s destruction regardless of the probing depth stated at the moment of the examination.

In the light of the above findings, we can confirm that there might be an association between pancreatic cancer and *NLRP3* polymorphism and, respectively, chronic pancreatitis and *NLRP2* polymorphism. Similarly, there is a distinct correlation between periodontitis and rs35829419 C>A in *NLRP3* polymorphism. Despite the fact that the authors of the study did not succeed in proving the statistical relevance of the above due to a small number of patients included in the investigated group, the obtained results (*p* = 0.107; OR = 3.3939) indicate a need to increase the number of patients with pancreatic cancer. The selected group constitutes approximately 10 % of all patients with pancreatic adenocarcinoma qualified for surgery.

The concept that a change in the composition of human saliva might be a sign of pancreatic cancer was first introduced by Zhang et al. (2010). In his study, he examined transcriptional genetic markers. The markers selected for the study (*Ki*-*RAS, DPM1, and ACRV1*) showed sufficient specificity and sensitivity to let us hope for their implementation in the future clinical practice. However, this evaluation procedure is quite costly and may only be conducted in laboratories with relevant equipment. Real-time PCR is an expensive procedure, and many hospitals have not yet introduced this technique for diagnostic purposes (Schefe et al. 2006). Chronic pancreatitis evinces a high comorbidity with alcoholism, which is also a risk factor for periodontitis. In order to rule out a possible interaction between alcohol abuse and pancreatitis, which both impact the state of the periodontium, we looked for though as yet failed to show any association between alcohol consumption and clinical parameters (BI, Offenbacher’s scale). Therefore, any worsening in the condition of oral cavity, which we discovered in patients with chronic pancreatitis, results from the inflammation alone, not from alcohol consumption.

Further research is needed in order to elucidate conditions of the pancreas both from the etiopathogenetic and the diagnostic points of view. What might be definitely stated at this stage of studies is that the line of action to be taken should span the molecular and genetic bases of pancreatic cancer and chronic pancreatitis. New insights into pathogenesis of these diseases might in turn lead to elaborating new molecular diagnostic methods. In our study, we proposed a novel approach to the problem—starting from the inflammatory basis, namely from the proteasome function, we searched for a possible connection between its activity (modulated by SNPs) and a very specific clinical situation, down to the personal level of the patient.

The conducted study did not fully confirm the hypothesis we put forth at the beginning of this paper in that a specific genotype is in fact associated with the periodontitis in each of the three investigated groups. This was due to a limited number of patients available for the study. An additional obstacle we encountered during our research was the heavy depression that the pancreas cancer patients suffer from. Moreover, a certain number of those patients suffer from multiple surgery complications such as pseudocysts and hematomas, which additionally contribute to deteriorating their overall health condition, which further on makes it even more difficult to encourage such a patient to participate in a scientific study and obtain a written informed consent from them.

Our study could contribute to enriching the findings of periodontal medicine as the overall state of oral cavity is the barometer of general health condition of a given individual. An introduction of a standard dental procedure consisting in a periodontal examination of the loss of CAL, bleeding index in patients with chronic pancreatitis, and pancreatic cancer risk factors could perhaps be worth considering in the future especially in the age group of 65–80 years at the highest risk of developing these conditions.

In conclusion, the study revealed that the *NLRP2* polymorphism was associated with chronic pancreatitis, whereas the *NLRP3* polymorphism was comorbid with pancreatic cancer. Similarly, the study demonstrated a clear and distinct correlation between rs35829419 C>A in NLRP3 and rs17699678 C>T in NLRP2 polymorphism and periodontitis in the form of an increased CAL index. The medical value of the conducted study thus lies in the fact that the in vitro findings were successfully confirmed by our clinical study.
